# Embryonic methionine triggers post-natal developmental programming in Japanese quail

**DOI:** 10.1007/s00360-024-01542-8

**Published:** 2024-03-23

**Authors:** Sawadi F. Ndunguru, Gebrehaweria K. Reda, Brigitta Csernus, Renáta Knop, Gabriella Gulyás, Csaba Szabó, Levente Czeglédi, Ádám Z. Lendvai

**Affiliations:** 1 Department of Animal Science, Faculty of Agricultural and Food Sciences and Environmental Management, Institute of Animal Science, Biotechnology and Nature Conservation, University of Debrecen, Debrecen, 4032 Hungary; 2 Doctoral School of Animal Science, University of Debrecen, Debrecen, 4032 Hungary; 3 Department of Evolutionary Zoology and Human Biology, University of Debrecen, Debrecen, 4032 Hungary; 4 Department of Animal Nutrition and Physiology, Faculty of Agriculture and Food Sciences and Environmental Management, University of Debrecen, Debrecen, 4032 Hungary

**Keywords:** l-methionine, IGF1, mTOR, Growth, Amino acid

## Abstract

Embryonic development is one of the most sensitive and critical stages when maternal effects may influence the offspring’s phenotype. In birds and other oviparous species, embryonic development is confined to the eggs, therefore females must deposit resources into the eggs to prepare the offspring for the prevailing post-natal conditions. However, the mechanisms of such phenotypic adjustments remain poorly understood. We simulated a maternal nutritional transfer by injecting 1 mg of l-methionine solution into Japanese quail eggs before the onset of incubation. The increase in early methionine concentration in eggs activated the insulin/insulin-like signalling and mechanistic target of rapamycin (IIS/mTOR) signalling pathways and affected post-natal developmental trajectories. Chicks from methionine-supplemented eggs had higher expression of liver *IGF1* and *mTOR* genes at hatching but were similar in size, and the phenotypic effects of increased growth became apparent only a week later and remained up to three weeks. Circulating levels of insulin-like growth factor-1 (IGF-1) and expression of ribosomal protein serine 6 kinase 1 (*RPS6K1),* the *mTOR* downstream effector, were elevated only three weeks after hatching. These results show that specific nutritional cues may have phenotypic programming effects by sequentially activating specific nutrient-sensing pathways and achieving transgenerational phenotypic plasticity.

**Supplementary Information:**

The online version contains supplementary material available at 10.1007/s00360-024-01542-8.

## Introduction

The early conditions experienced by animals are important in understanding the causes of phenotypic variation (Flatt 2001; Meillère et al. 2015; Monaghan 2008). One key component contributing to early phenotypic variation is the maternal investment (Lindström 1999). Mothers can transfer various signals and cues to the offspring to adjust their phenotype and fitness to the current environmental conditions. However, the importance of such transgenerational adaptive plasticity for phenotypic evolution remains debated (Mousseau and Fox 1998; Reed and Clark 2011; Williams and Groothuis 2015). Recently, it has become clear that embryonic development is one of the critical stages when maternal effects may influence the offspring’s phenotypic and physiological responses (Groothuis et al. 2019; Reed and Clark 2011) and embryos have evolved the mechanisms to incorporate maternal signals for their adaptive performance (Groothuis et al. 2020; Stier et al. 2020).

In birds and other oviparous species, embryonic development is confined to the eggs. Eggs are “sealed capsules” where the mother needs to deposit all the transmitted factors such as nutrients, hormones, vitamins, carotenoids to influence offspring development and phenotype (Blount et al. 2002; Groothuis et al. 2019; Mentesana et al. 2021). However, the deposition of maternal resources into the egg depends on various conditions. For instance, habitat type and quality, perceived predation risk or social environment may affect the allocation of resources into the eggs (Jones et al. 2014; Sharda et al. 2021; Séchaud et al. 2022), which may explain offspring fitness (Lahaye et al. 2015).

One of the major ways maternal conditions influence individuals’ phenotype is through the programming effects of yolk hormones (Groothuis and Schwabl 2008; Groothuis et al. 2019; Martin and Schwabl 2008). The maternally deposited hormones are important in mediating the offspring’s phenotypes as they provide signals for adaptive growth conditions (Groothuis and Schwabl 2008). One of the hormonal systems that most directly influences development is the insulin/insulin-like signalling pathway (IIS) (Regan et al. 2020). IIS is a network known to respond to the nutritional status of the organism and regulate metabolism, growth, and development (Schwartz and Bronikowski 2016). In vertebrates, insulin-like growth factor 1 (IGF-1) is the major ligand of this pathway that controls embryonic and postnatal growth and development by stimulating cell proliferation, migration, differentiation, and protein synthesis (Beccavin et al. 2001; Luisi et al. 2012; Iresjö et al. 2022; Regan et al. 2020). Despite its fundamental importance (Lodjak and Verhulst 2020), the IIS pathway remains poorly studied in birds.

The IIS system does not work in isolation in regulating energy metabolism; it is directly integrated with its downstream effector, the mechanistic target of rapamycin (mTOR) (Braun and Sweazea 2008; Fernandes and Demetriades 2021; Szwed et al. 2021). The mTOR is an evolutionarily highly conserved nutrient sensing pathway that integrates intracellular signals from nutrient availability and serves as a central regulator of cell metabolism and growth (Laplante and Sabatini 2009; Reda et al. 2024; Regan et al. 2020; Saltiel and Kahn 2001). One of the nutrient cues that specifically activate mTOR is methionine (Zeitz et al. 2019). Methionine is an essential amino acid that alone may influence life history traits. Methionine concentration has a positive association with reproduction but a negative association with longevity (Mota-Martorell et al. 2021; Zou et al. 2020). However, when amino acids are in a balanced state, methionine has been observed to exert a positive influence on both fecundity and longevity (Grandison et al. 2009).

Deposited egg amino acids therefore may also serve as potential maternal signals through which the IIS/mTOR pathway can influence embryonic and post-hatch development and have significant effects on the phenotype and performance fitness of the offspring (Giordano et al. 2014; Ronget et al. 2018). While the maternal programming effects of growth hormones are well documented, whether a non-hormonal, nutritional amino acid can influence developmental trajectories through the activation of the IIS/mTOR remains uncertain. For example, embryonic supplementation of sulfur-containing amino acids (methionine plus cysteine) in chicken has been shown to improve embryonic development, circulating IGF-1 and intestinal growth of newly hatched chicks exposed to heat stress (39.6 °C) for 6 h per day during incubation (Elwan et al. 2019). However, the mechanisms behind these effects and whether they translate into post-natal phenotypic differences remain unknown.

We hypothesized that this nutritional cue would activate molecular mechanisms in the nutrient-sensing pathway that influence phenotypes. Therefore, we determined circulating levels of IGF-1, and hepatic expression of growth-related genes (*IGF1*, *mTOR* and a downstream effector of mTOR, the ribosomal protein serine 6 kinase 1, *RPS6K1*) that influence the early phenotypic development in the offspring.

## Materials and methods

### Experimental design

We collected freshly laid Japanese quail (*Coturnix japonica*) eggs from a total of 63 females of the same age group (18 month) and kept them at room temperature between 16 and 18 °C for 1–5 days. On the day of laying, we weighed the eggs on a digital scale (± 0.01 g) and separated eggs of similar mass (11.0 ± 0.5 g), to eliminate the effect of differences in egg mass. When we amassed a total of 200 eggs, we randomly selected half of them, to which we injected 1 mg L-methionine dissolved in 50 μl saline solution, while the remaining eggs (controls) received physiological saline solution. Our objective was to make only a slight increase in the methionine concentration *in ovo.* Methionine amino acid concentration in whole egg was quantified in duplicates, from a sample of 100 g pooled eggs (based on *n* = 12 eggs) at the accredited Central Laboratory of the Agriculture and Food Products, Faculty of Agricultural and Food Sciences and Environmental Management, University of Debrecen, Hungary. The whole egg methionine concentration was 0.42 m/m %, which closely mirrored the value documented in the extensively studied Japanese quail egg contents by the US Department of Agriculture (0.421 m/m %, USDA 2019). Given the average weight of 11.0 g of the eggs in our experiment, the 1 mg of extra L-methionine increased its concentration by 2.16% above the average in Japanese quail eggs and this post manipulation value was still in the natural range of egg methionine concentrations reported by earlier publications (0.43 m/m %, Genchev 2012; 0.43 m/m%, Tolik et al. 2014; 0.421 m/m %, USDA 2018).

Before the injections, we prepared a batch of the amino acid solution by dissolving crystalline L-methionine (CAS No. 63-68-3, Sigma Aldrich, BioUltra, > 99.5%) in 0.9% physiological saline solution (Sigma Aldrich). Saline and amino acid solutions were sterilized by autoclaving. Before the injection, we disinfected the broad end site of the egg with 70% ethanol, then we incised a hole using a sterile 26G needle. We then injected 50 μl of either the L-methionine or saline solution into the egg yolk using a 50 μl ethanol sterilized Hamilton syringe. Finally, we sealed the egg's injection point with candle wax. We performed all injections before the incubation started on embryonic day zero.

Immediately after the injections, we transferred the eggs to an automatic incubator (WQ-63 Model 2021 Version 2, AGROFORTEL, Budapest Hungary) setting conditions to 37.8 ± 0.5 °C and 50–60% relative humidity. On day 8 of the embryonic development, we candled the eggs with a flashlight, and removed those where embryonic development had not started or had stopped. Egg freshness is a major determinant in hatchability. However, studies have shown that egg quality and freshness is reduced only after 7–10 days (Drabik et al. 2021; Tan et al. 2020) and therefore recommend incubating eggs collected within a week of laying (Abioja et al. 2021; Fasenko 2007; Goliomytis et al. 2015). To be more conservative, we only used eggs up to five days after laying. We did not consider egg age as a factor that may have induced embryo mortality. Since the experiment only used those eggs that hatched successfully and the treatment was assigned randomly, it is unlikely that any variation of egg age could have confounded our results. Moreover, we suppose that treatment could have caused selective mortality in the eggs of the slow growing embryos. We observed that 68% from the control and 61% from methionine-injected group showed embryonic development during candling on day 8. Although hatching success tended to be lower in the methionine-injected group compared to the control one (31.15% vs. 51.47% hatched of developing embryos), this difference remained non-significant (${x}^{2}$ = 1.80, *df* = 1, *P* = 0.176). On day 14 of incubation, we transferred the eggs from the incubator trails to the hatching tray and reduced the temperature to 37.5 °C and increased relative humidity to 65–70%. We checked the hatching process every 12 h for hatching events and hatchability of the fertile eggs.

### Rearing experimental hatchlings

We transferred immediately the hatchlings from each experimental group to two separate cages based on their treatment (40 cm long × 50 wide × 40 cm height) and reared for three weeks (21 days). We provided free access to unlimited feed nutrient content according to (National Research Subcommittee on Poultry Nutrition and Subcommittee on Poultry Nutrition National Research Council, 1994) and water. We kept all hatchlings under uniform standard management conditions throughout the experimental period. We recorded post-hatch body mass using an electronic scale (± 0.01 g), and tarsal, head, and wing length were measured with a vernier calliper (± 0.01 mm) on day 1, 3, 5, 7, 14, and 21. All measurements were taken by the same person who was unaware of the treatment subjected to experimental groups. Given that Japanese quails lay one egg daily, and that only 51 total hatchlings were included in the study from 63 hens, most eggs are expected to be originated from different females, yet some chicks likely shared the same parents. Since all parents in the experiment arrived from the same breeding stock, birds in our study (both adults and chicks) are likely to genetically relate to various degrees. To fully accounting for the genetic relatedness requires a full pedigree, which was unavailable. Maternal identity of the eggs was not recorded, and juvenile individuals were thus treated as independent data points.

### Sample collection

**Fig. 1:**
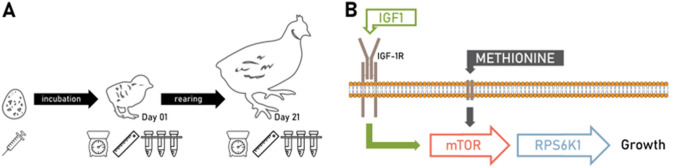
**A** Schematic representation of the experimental protocol. A syringe icon indicates the L-methionine manipulation before incubation; the lab scale, the ruler and the centrifuge tubes represent body mass measurements, morphological measurements, and blood plus tissue sampling, respectively. **B** Simplified diagram showing the investigated elements of the nutrient sensing pathway (*IGF1*: insulin-like growth factor 1, *mTOR*: mechanistic target of rapamycin, *RPS6K1*: ribosomal protein serine 6 kinase 1)

We randomly selected a total of 8 quail chicks from each experimental group for blood and tissue sampling post hatching after recording body mass (Fig. 1). Tissue and blood sampling was done at one time point on day 1 and 21 post hatching. Body mass was recorded at variable days for the remaining chicks until 21 days when we collected the second blood and tissue samples. We collected blood samples (~ 65 µl for day-old and ~ 80 µl for 21 days old birds) into heparinised capillary tubes by venepuncture using a 26G sterile needle. We immediately centrifuged the plasma samples and separated from red blood cells using a Hamilton syringe and stored at − 20 °C for further analyses. To reduce the variation in circulating IGF-1 levels in 24 h, blood samples on day 21 were collected at the same time as the first samples on day 1 (Lendvai et al. 2021). We sacrificed birds by cervical dislocation after blood collection; collected liver samples and snap-froze in dry ice, and then store at − 80 °C for further analysis. Liver is a multi-purpose vital organ responsible for many metabolic functions including nutrient homeostasis, protein, carbohydrate, fat, vitamins and minerals metabolism, hormonal synthesis (Xu et al. 2019). Changes in nutrients status are easily adapted in the liver. During the postnatal development many changes in the liver lead to differential functions of the liver at different developmental stages in broiler chickens (Lee et al. 2012; Payne et al. 2019; Xu et al. 2019). Changes in hepatic gene expression take place during prenatal and postnatal developmental stages to support the wide range of metabolic functions to influence trait fitness (Lee et al. 2012).

### ELISA

We measured circulating plasma IGF-1 levels by a competitive enzyme-linked immunosorbent assay (ELISA) as described previously (Mahr et al. 2020). We analysed samples in duplicates (*n* = 64) on a single plate and the intra-assay coefficient of variation (CV) was 9.8%.

### Real-time PCR

We extracted total RNA from frozen liver tissue using TRIzol reagent and Direct-zol™ RNA MiniPrep (Zymo Research; Orange, CA, USA) according to the manufacturer's protocol, including DNA digestion step. We checked RNA integrity by 1.5% agarose gel electrophoresis, then determined the RNA concentration (ng/µl) and percentage purity (%) spectrophotometrically with SYNERGY/HTX multi-mode plate reader (BioTek Instruments Inc, USA). We performed reverse transcription using the qScript cDNA synthesis kit (Quantabio Reagent Technologies; QIAGEN Beverly Inc., USA) in a 20 μl final volume containing 5 × cDNA supermix, 200 ng RNA template and distilled water, using PCRmax Alpha thermal cycler (Cole-Parmer Instrument Co. Ltd., UK). Conditions consisted of reverse transcription 25 °C for 5 min, 42 °C for 30 min and 85 °C for 5 min. We designed intron-spanning forward and reverse primers for quail using the Oligo 7 software and checked for target identity using Primer-Blast software of the National Centre for Biotechnology Information (NCBI) (http://www.ncbi.nlm.nih.gov, supplementary material Table S2). We performed quantitative PCR in Agilent AriaMx Real-time PCR System (Agilent Technologies, USA) and applied 5 × HOT FIREPol^®^ EvaGreen^®^ qPCR Mix Plus (Solis BioDyne; Tartu, Estonia), 2 ng cDNA template, 200 nM of each primer, and distilled water in a 10 μl final volume of each sample. We ran the samples in duplicates.

We normalised relative changes in gene expression against *RPL19* gene expression as the most stabile reference gene selected from 6 housekeeping genes such as *ACTB*, *GAPDH*, *RPL19*, *RPS8, 18S*, and *RPL13* by 3 algorithms (delta Ct, Best Keeper, NormFinder) (Simon et al. 2018; Vitorino Carvalho et al. 2019). Relative gene expression compares the expression level of gene of interest or the target gene to the expression level of the reference gene housekeeping genes that are usually expressed relatively constant in all cells and most of the conditions (Joshi et al. 2022). We collected raw data with Aria AgilentMx 1.8 software. We calculated relative gene expression of the target genes (*mTOR*, *RPS6K*1, and *IGF1*) in fold change using the double-ΔCT method (Schmittgen and Livak 2008). We considered the sample with the highest delta Ct value as calibrator to calculate the double-ΔCT value (Pabinger et al. 2014).

### Statistical analyses

We carried out all statistical analyses and made inferences using R-version 4.0.3 software (RStudio Team 2020). We used a linear model to analyze the effects of IGF-1 levels as a response variable while treatment and days as independent variables. We analysed growth in terms of increasing body mass and development of morphological traits (wing, head, tarsal, and feather lengths) and body condition using scaled mass index (Peig and Green 2009) across the days (1, 3, 5, 7, 14, and 21) in a linear mixed model with body mass and morphological traits as dependent variables, treatment, and days as fixed factors, while individual bird identity as random factor. We also fitted a fixed factor linear model to analyse the effects of treatment on the relative gene expression of *mTOR*, *IGF1*, and *RPS6K1* as a response variable with treatment and day as fixed factors. We used estimated marginal means to compare body mass/morphological traits and relative gene expression between the treatment groups within each day using the ‘emmeans’ package and significance p-values were adjusted based on tukey method package (Searle et al. 1980).

## Results

### Body mass and morphological traits

**Fig. 2:**
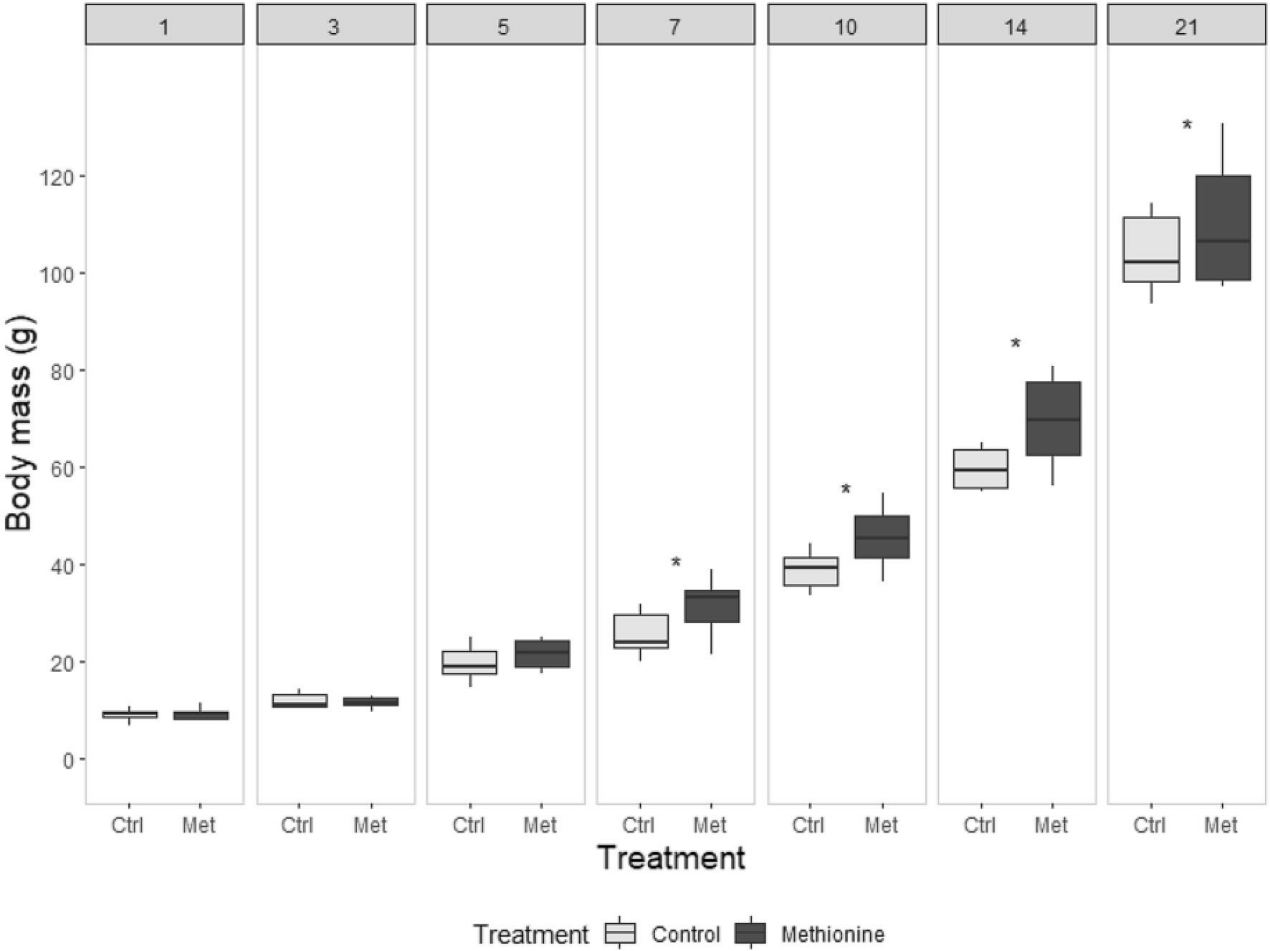
L-methionine injection in eggs speeds up post-hatch body mass gain in Japanese quail chicks (see Table S3 for detailed information on sample size). The thick line indicates the median, the box shows the interquartile range, and the whiskers extend to the minimum and maximum values. ‘Ctrl’ and ‘Met’ refer to the control and methionine-injected groups, respectively. Asterisks denote significant differences (*P* < 0.05) among the treatment groups at each time point and numbers at the top of each panel indicates the age of the chicks (days)

The body mass at hatching through day 5 was not different between treatment groups. However, chicks in the methionine-injected group grew faster (treatment × age interaction, *P* < 0.001), starting from day 7, their body mass was consistently higher than controls till day 21 (Fig. 2, Table S3). Wing and head length did not differ between treatments through all days (Fig. S1–3) respectively while tarsus length became only significant on day 21 (*P* = 0.033, Fig. S3). Body condition did not differ, as the scaled mass index remained statistically non-significant) between the control and treatment groups throughout the study (*P* > 0.05).

### IGF-1 levels

**Fig. 3:**
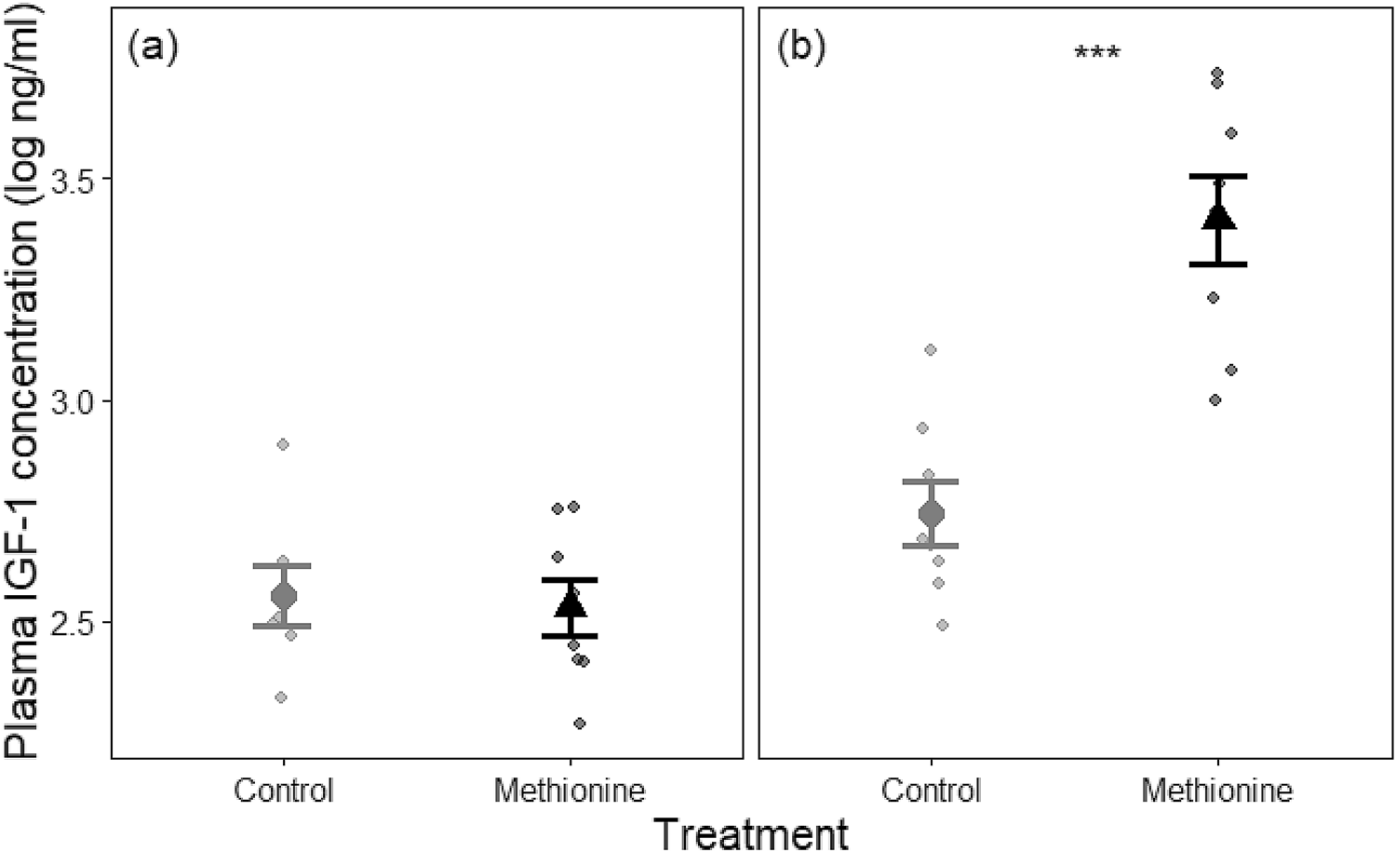
L-methionine injection in eggs increased post-hatch circulating levels of IGF-1 in Japanese quail chicks (*n* = 8 in each group). Asterisks*** denote significant differences between the treatment groups at *P* < 0.001, panels indicate age of the chicks (**a**) 1-day old chicks and (**b**) 21-days old chicks. The big circles or triangles and error bars indicate mean ± standard error, the small circles show individual measurements

IGF-1 levels increased with the age of birds, but in a treatment-specific manner. IGF-1 levels on day 1 did not differ significantly between the methionine-treated and control chicks (*P* = 0.964, Fig. 3a). In contrast, three weeks later, at 21 days, while all chicks had higher IGF-1 levels than after hatching (*P* < 0.001), methionine-treated birds had significantly increased their IGF-1 levels more than the control group (*P* < 0.001, Fig. 3b).

### Relative gene expression

**Fig. 4:**
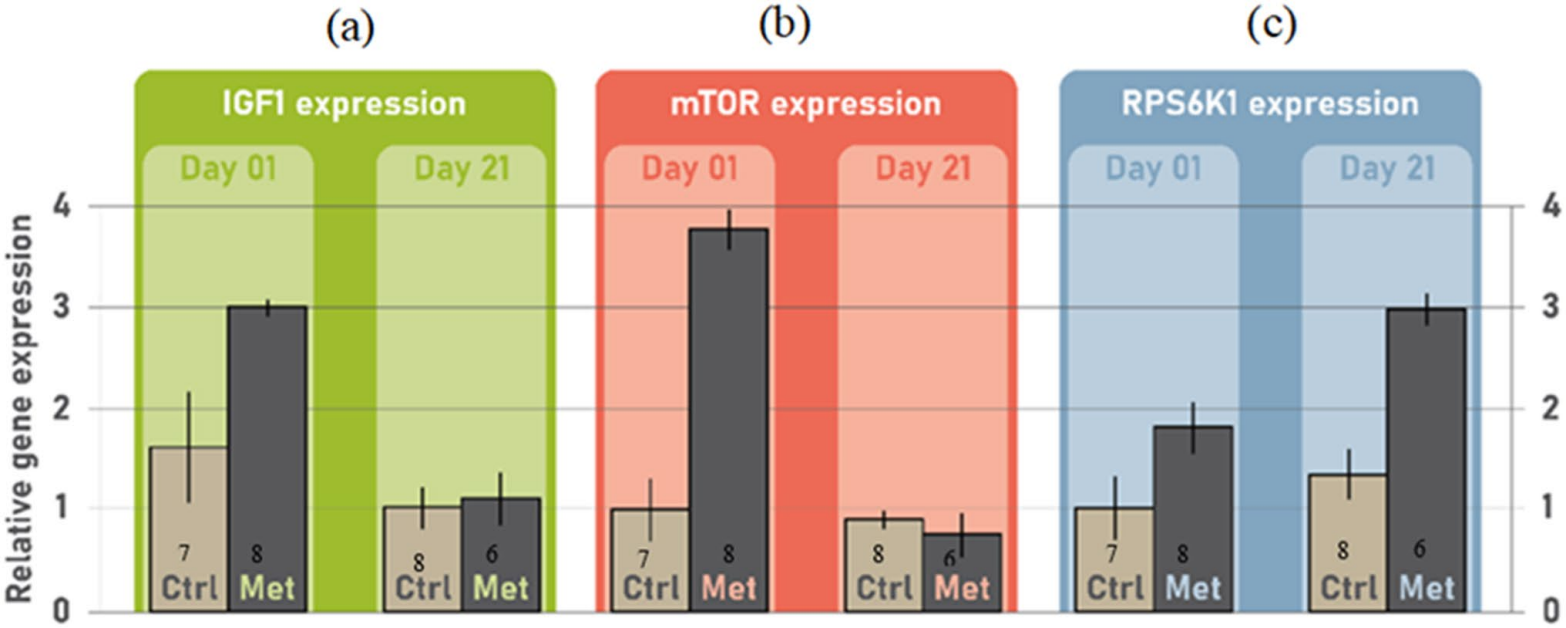
Gene expression patterns (mean ± SE) of insulin-like growth factor 1 (*IGF1*), mechanistic target of rapamycin (*mTOR*) and ribosomal protein serine 6 kinase 1 (*RPS6K1*). L-methionine injection increased the relative gene expression of (**a**) *IGF1* on 1 day post hatching, (**b**) *mTOR* on day 1 post hatching, and the relative gene expression of (**c**) *RPS6K1* in 21 days old Japanese quail chicks. Numbers on each column indicate sample size per group

The relative gene expression of *IGF1, mTOR,* and *RPS6K1* were influenced by L-methionine treatment. On day-old chicks, relative gene expression of *IGF1* significantly increased in the methionine-treated group compared to the controls (*P* = 0.005), however, by day 21, the relative gene expression of *IGF1* became similar between the groups (*P* = 0.998, Fig. 4a). Further, methionine treatment also increased *mTOR* expression in day-old chicks (*P* = 0.001), which disappeared by day 21 (*P* = 0.993, Fig. 4b). However, while treatment groups did not differ in their initial *RPS6K1* expression in day-old chicks (*P* = 0.164), by day 21 methionine-treated chicks expressed more *RPS6K1* than controls (*P* < 0.001, Fig. 4c).

## Discussion

The essential amino acid, L-methionine is a key trigger for the mTOR signalling pathway that has a major impact on life history as it regulates cellular and organismal growth, reproduction, and ageing (Kitada et al. 2020; Zhou et al. 2016). Here, we present evidence for the first time, that increase in yolk L-methionine concentration influences the IIS/mTOR signalling pathways and affects post-natal developmental trajectories in a bird species.

Our manipulation simulated an early maternal transfer of nutritional L-methionine, as injections were made before the onset of incubation. Since eggs were incubated and chicks raised under standardized conditions, any difference between the treatment groups must be related to the L-methionine supplementation. This mild increase in L-methionine however, altered the developmental trajectory of the chicks, with different elements manifesting at different time points. At hatching (day 1) treatment groups had equivalent body mass and biometric measurements. However, already at this stage, the treatment induced significant gene expression signature of *IGF1* and *mTOR*. Despite the early upsurge of *mTOR/IGF1* expression, circulating levels of IGF-1 at hatching was similar between the treatment groups, and body mass and skeletal growth also remained identical up to a week. However, starting from day 7, chicks hatched from the methionine-injected group consistently increased body mass more than the control. While this difference in body mass lasted until the end of the experimental period, the methionine treatment did not induce a significant difference in skeletal measurements (wing, head length and tarsus length), except for tarsus that by day 21 also became significantly larger in the methionine treated group. Despite the birds in the treatment group becoming heavier, the body condition, as measured by the scaled mass index, remained consistent and statistically non-significant compared to the control group throughout the study. This result indicates that chicks in the methionine group did not gain a significantly greater proportion of body mass compared to the control group, implying that the treatment induced both increased skeletal growth and body mass gain, albeit the latter becoming apparent sooner.

Three weeks after hatching, we saw a reorganization of the physiological and gene expression pattern. At this time, plasma IGF-1 levels and *RPS6K1* gene expression increased in L-methionine-treated individuals compared to controls, while *mTOR* and *IGF1* gene expression were no longer different between the treatment groups. These unmatched protein and gene expression suggest that proteins may be post-transcriptionally regulated, such as stability of mRNA or elevated half-life of the protein resulting from the post-translational modifications often altering the protein levels (Csernus et al. 2023; Ideker et al. 2001). After hatching, an increased hepatic *IGF1* mRNA expression may lead to increased production of IGF-1 protein. However, there can be delays in the translation of mRNA into protein and post-translational modifications that affect the secretion and stability of the IGF-1 protein (Gedeon and Bokes 2012). This could explain the lag in the elevation of circulating IGF-1 levels. Even once hepatic *IGF1* mRNA expression returned to control levels, other tissues or organs may have responded to the manipulation by producing more IGF-1, contributing to the delayed elevation in plasma levels. Nestlings at one day old and nestlings at 21 days old may have different physiological states and developmental stages. These differences could affect how their bodies respond to the manipulation and regulate IGF-1 levels.

Our findings align with the concept that nutrient developmental programming during critical development windows can have short-term and longer-term consequences in the offspring (Andrieux et al. 2022; Buchanan et al. 2022). The increased *mTOR* and *IGF1* gene expression on day 1 indicates that these genes are important during the embryonic and early post-natal growth and development, the effects mediated by methionine manipulation. Subsequently, plasma IGF-1 levels and *RPS6K1* gene expression appeared to be more important during the late post-natal development as they were increased at the juvenile stage. Principally, the evidence reveals that mTOR mediate essential roles during the embryonic development and early postnatal, with *RPS6K1* controlling the physiological reorganisation during the post-natal growth and development. While body mass and circulating levels of IGF-1 measured late during the postnatal period was increased, it suggested that methionine programming may improve further growth and development through the enhancement of IGF-1 synthesis (Wan et al. 2017; Wen et al. 2017). The fact that circulating IGF-1 activity increases towards developmental stages of birds (Lodjak et al. 2018; Lodjak and Verhulst 2020), could be the reason to explain why the effects of our treatment on body mass was delayed and became only apparent a week later post-hatching. This is supported by the lack of significant difference in body mass between the chicks at day 1 of the same age, but also at different developmental stages of first week and apparently significant a week later. Our findings coincide with a study in chicken that reported a lack of significant increase in body mass of a day-old broiler chicks hatched from eggs injected with L-methionine during the late embryonic stage (Chen et al. 2021). Although in our study, the treatment did not induce faster growth at hatching, recent studies showed that L-methionine supplemented to day-old broiler chicks stimulated faster growth and development (Akter et al. 2020; Shen et al. 2015). While methionine supplementation at an early stage of growth from day 3 to 6 in blue tit and magpie nestlings impaired growth (Brommer 2004; Soler et al. 2003), in contrast, methionine supplementation in great tit nestlings from day 9 did not alter their growth rate (Wegmann et al. 2015). The weaker effect of growth in body size in nestlings may demonstrate the casual relationship between IGF-1 and early postnatal growth where IGF-1 levels were significantly higher in 7 days old than 13 days old in pied flycatcher nestlings (Lodjak et al. 2018).

Our results point to a nutritional mechanism through which maternal effects may be manifested. However, the extent to which mothers utilize this mechanism, the degree of control they exert over methionine deposition and the varying sensitivity of different species to this form of maternal influence remain intriguing questions awaiting further exploration. Current evidence from field studies suggests that the differences in food supply may induce variation plasma amino acids levels. For instance, Herring gulls (*Larus argentatus*) from different geographical locations exhibited different levels of plasma methionine compared to the reference value (Hebert et al. 2002). The variations of the plasma amino acids in gulls are possibly linked to the geographical variations of protein availability which may be influenced by prey availability. Additionally, increased plasma concentrations of non-essential amino acids and decreased essential amino acids were associated with egg formation (Taylor et al. 1970). Birds may prioritize early maternal allocation by adjusting amino acids deposition into eggs based on the environmental factors such as habitat quality, predation risk and social environments (Dixit et al. 2017; Macelline et al. 2021; Mori et al. 2020). These cues may shape early maternal allocation strategies through specific amino acid deposition into the egg such as methionine, serving as energy budget for the offspring growth and development (Fontaine and Martin 2006).

The results of this experiment shed light on the mechanisms by which early maternal investment can affect the offspring phenotype. To control growth and development of the embryos and subsequent offspring, mothers allocate non-genetic resources including nutrients in the eggs (Reed and Clark 2011). While earlier studies have shown how early maternal investment in eggs can impact the offspring’s fitness (Price 1998; Valcu et al. 2019), the major route of maternal programming was thought to be the direct transfer of hormones into the eggs (Darras 2019; Groothuis et al. 2019; Kumar et al. 2019). Here, we show that nutritional cues may also enhance postnatal growth and development through specific nutrient-sensing pathways.

## Conclusion

We have shown that a mild increase in L-methionine before the onset of embryonic development upregulated *mTOR/IGF1* gene expression at hatching, which resulted in significant increase in growth only after a week post-hatching. This single, initial amino acid cue resulted in increased IGF-1 hormonal level and *RPS6K1* gene expression even three weeks post-hatching. These results show that maternally derived nutritional cues may have powerful programming effects on post-natal developmental trajectories with maintained increased body mass showing that it is responded for growth and development during the post-natal period and highlights the importance of amino acids as maternal signals to promote transgenerational phenotypic plasticity in birds.

### Supplementary Information

Below is the link to the electronic supplementary material.

Supplementary file1 (XLSX 11 KB)

Supplementary file2 (XLSX 10 KB)

Supplementary file3 (XLSX 6 KB)

Supplementary file4 (TXT 4 KB)

Supplementary file5 (TXT 4 KB)

Supplementary file6 (TXT 4 KB)

Supplementary file7 (DOCX 65 KB)

## Data Availability

The authors confirm that the data supporting the findings of this study are available within the article as supplementary materials.
