# Supplementary material for: Embryonic methionine triggers post-natal developmental programming in Japanese quail
Source: J Comp Physiol B. 2024 Mar 23;194(2):179–89. doi: 10.1007/s00360-024-01542-8 (PMC11070397; doi:10.1007/s00360-024-01542-8)
Supplement: Supplementary file 7 — Supplementary file7 (DOCX 65 KB) [file 360_2024_1542_MOESM7_ESM.docx]

**Embryonic methionine triggers post-natal developmental programming in Japanese quail**

Sawadi Fransisco Ndunguru^1,2,3*^, Gebrehaweria Kidane Reda^1,2,3^, Brigitta Csernus^3^, Renáta Knop^1^, Gabriella Gulyás^1^, Csaba Szabó^4^, Levente Czeglédi^1^, Ádám Zoltán Lendvai^3*^

*^1^Department of Animal Science, Institute of Animal Science, Biotechnology and Nature Conservation, Faculty of Agricultural and Food Sciences and Environmental Management, University of Debrecen, 4032 Debrecen, Hungary*

*^2^Doctoral School of Animal Science, University of Debrecen, 4032 Debrecen, Hungary*

*^3^Department of Evolutionary Zoology and Human Biology, University of Debrecen, 4032 Debrecen, Hungary*

*^4^Department of Animal Nutrition and Physiology, Faculty of Agriculture and Food Sciences and Environmental Management, University of Debrecen, 4032 Debrecen, Hungary*

*****Correspondences**:** **[ndunguru@agr.unideb.hu](mailto:ndunguru@agr.unideb.hu),** [**az.lendvai@gmail.com**](mailto:az.lendvai@gmail.com)

**Journal of Comparative Physiology B**

**Appendices (As supplementary materials)**

**Table S1. Composition and nutrient level of the basal diet**

| **Feed ingredients** | **Composition %** |
| --- | --- |
| Corn | 23.69 |
| Wheat | 30.00 |
| Soybean meal (46% CP) | 34.85 |
| Fishmeal | 5.00 |
| Sunflower oil | 4.09 |
| Limestone | 1.01 |
| MCP | 0.37 |
| Salt | 0.24 |
| DL-Methionine | 0.10 |
| L-Threonine | 0.13 |
| Vitamin and mineral premix^a^ | 0.50 |
| **Nutrient composition** |  |
| Metabolisable energy MJ/kg | 12.13 |
| Crude protein | 24.00 |
| Calcium | 0.80 |
| available Phosphorus | 0.30 |
| Sodium | 0.15 |
| Methionine | 0.45 |
| Lysine | 1.34 |
| Threonine | 1.02 |
| Tryptophan | 0.29 |

^a^ 1 kg premix provided: 1000000 NE vitamin A, 200 000 NE vitamin D_3_, 4900 mg/kg vitamin E, 200 mg vitamin K_3_, 150 mg vitamin B_1_, 500 mg vitamin B_2_, 1200 mg Ca-d-Pantothetane, 400 mg vitamin B_6_, 2 mg vitamin B_12_, 11 mg biotin, 2502 mg niacin, 60 mg folic acid, 300000 mg choline cloride, 13200 mg Zn, 1920 mg Cu, 9612 mg Fe, 13200 mg Mn, 180 mg I, 42 mg Se, 12 mg Co.

**Table S2. Characteristics of the primer pairs**

| Gene | Gene name | Primer sequences (5’ → 3’)  (forward/reverse) | NCBI GenBank | Amplicon length (bp) | Tm (°C) |
| --- | --- | --- | --- | --- | --- |
| ACTB | Actin beta | F: CCCCTGAACCCCAAA GCCAAC | XM_015876619.1 | 114 | 63.78 |
|  |  | R: ACCAGAGGCATACAGGGACAGC |  |  | 64,05 |
| mTOR | mechanistic target of rapamycin | F: CCGAAGCATTGAATTGGCCCT | XM_015882433.2 | 116 | 61.57 |
|  |  | R: CATCTCTCAAAGGCAGCGGACC |  |  | 63.50 |
| RPS6K1 | ribosomal protein S6 kinase 1 | F: AGGCAGGAACCCTCCGTGCAA | XM_015883670.2 | 105 | 66.43 |
|  |  | R: AGCTCAAACTGCGAAGGGTCGG |  |  | 65.30 |
| IGF-1 | insulin-like growth factor-1 | F: CACTATGCGGTGCTGAGCTGGTT | XM_015867574.2 | 117 | 65.42 |
|  |  | R: TCCCCTTGTGGTGTAAG CGTCT |  |  | 63.60 |
| RPL19 | ribosomal protein L19 | F: CATCGGTAAGAGGAAGGGT | XM_015885843.1 | 162 | 55.80 |
|  |  | R: ACGTTGCCCTTGACCTTCAG |  |  | 60.54 |
| RPS8 | ribosomal protein S8 | F: CTGACACCTGAGGAAGAAGA | XM_015870342.1 | 195 | 56.20 |
|  |  | R: CTTGCCTTCCAACACGTAGC |  |  | 59.48 |
| RPL13 | ribosomal protein L13 | F: CCACAAGGACTGGCAGCG | NM_204999 | 135 | 61.05 |
|  |  | R: ACGATGGGCCGGATGG |  |  | 58.64 |
| 18S | ribosomal RNA | F: CGAAAGCATTTGCCAAGAAT | AF173612 | 98–99 | 55.52 |
|  |  | R: GGCATCGTTTATGGTCGG |  |  | 55.97 |

**Table S3.** Pairwise comparison of marginal means of body mass, standard error of means in the L-methionine-injected and control groups and related statistics (t-ratio and p-value) from a Tukey HSD post-hoc test.

|  | **L-methionine** | **Saline injected** | **t-ratio** | **p-value** |
| --- | --- | --- | --- | --- |
| **Day** | **Mean body mass (SEM)** | |  |  |
| Day 1 | 9.41(0.23) (n=35) | 9.120(0.140) (n=16) | -0.200 | 0.141 |
| Day 3 | 11.68(0.35) (n=8) | 12.010(0.056) (n=8) | 0.271 | 0.787 |
| Day 5 | 21.59(1.08) (n=8) | 19.550(1.310) (n=8) | -0.765 | 0.445 |
| Day 7 | 31.50(2.03) (n=8) | 25.625(1.590) (n=8) | -2.439 | 0.016 |
| Day 10 | 45.45(2.17) (n=8) | 39.050(1.320) (n=8) | -2.668 | 0.008 |
| Day 14 | 69.59(3.46) (n=8) | 59.860(1.460) (n=8) | -4.119 | 0.001” |
| Day 21 | 110.19(4.64) (n=8) | 104.060(2.740) (n=8) | -2.548 | 0.012 |


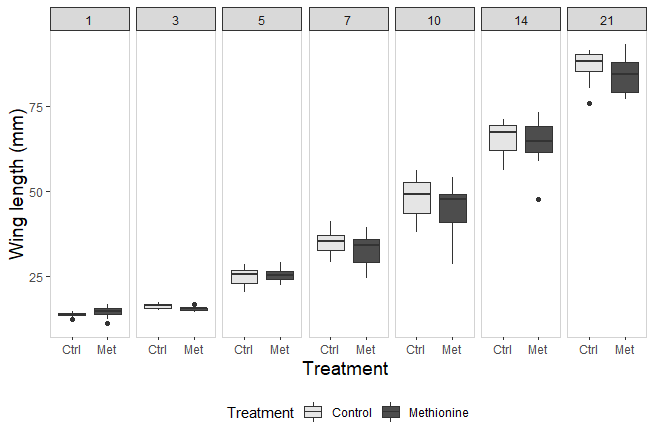


**Figure S1**. L-methionine injection had no effect on wing length of Japanese quail chicks.

(see table S3 for detailed information on sample size). The thick line indicates the median, the box shows the interquartile range, and the whiskers extend to the minimum and maximum values. ‘Ctrl’ and ‘Met’ refer to the control and methionine-injected groups, respectively. Asterisks denote significant differences among the treatment groups at each time point and numbers at the top of each panel indicates the age of the chicks.


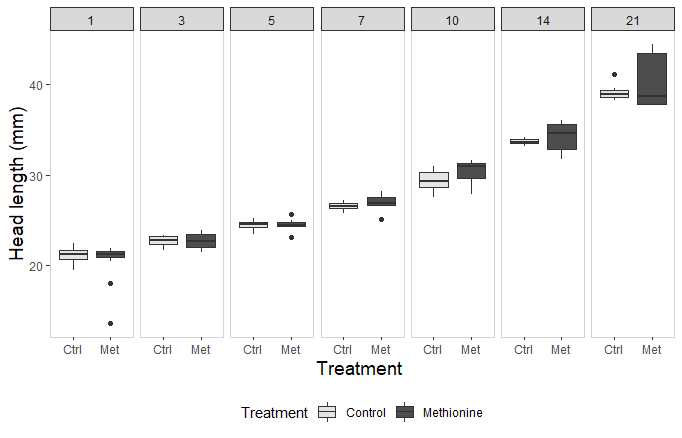


**Figure S2.** L-methionine injection had no effect on head length of Japanese quail chicks. *(see* table S3 *for* *detailed information on sample size). The thick line indicates the median, the box shows the interquartile range, and the whiskers extend to the minimum and maximum values. ‘Ctrl’ and ‘Met’ refer to the control and methionine-injected groups, respectively. Asterisks denote significant differences among the treatment groups at each time point and numbers at the top of each panel indicates the age of the chicks.*


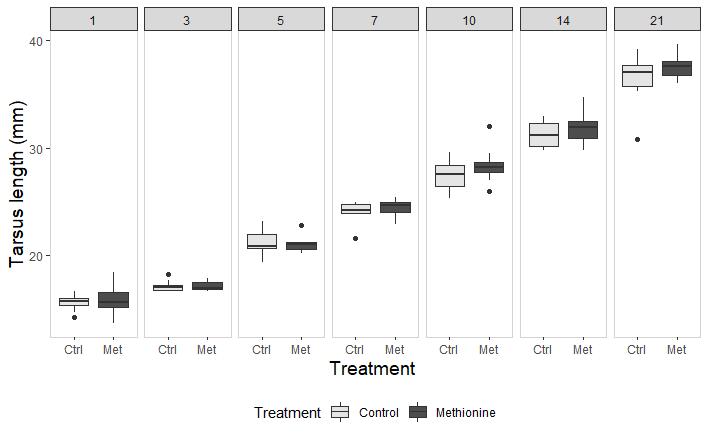


**Figure S3.** L-methionine injection had no effect on tarsus length Japanese quail chicks. *(see* table S3 *for* *detailed information on sample size). The thick line indicates the median, the box shows the interquartile range, and the whiskers extend to the minimum and maximum values. ‘Ctrl’ and ‘Met’ refer to the control and methionine-injected groups, respectively. Asterisks denote significant differences among the treatment groups at each time point and numbers at the top of each panel indicates the age of the chicks.*
